# *MYL1*‐Related Congenital Myopathy: Clinical, Genetic and Pathological Insights

**DOI:** 10.1111/nan.70025

**Published:** 2025-06-09

**Authors:** Irene Madrigal, Cristina Villar‐Vera, Gemma Arca, Jesica Expósito‐Escudero, Laia Rodríguez‐Revenga, Andres Piolatti‐Luna, Nuria Muelas, Roger Vilchez, Maria Ciutad Celdran, Anna Codina, Berta Estévez‐Arias, Laura Carrera‐Garcia, Carlos Ortez, Leonardo Rodriguez‐Carunchio, Giorgia Sebastiani, Inmaculada Azorin, Andrés Nascimento, Cristina Jou, Juan Jesus Vilchez, Daniel Natera‐de Benito

**Affiliations:** ^1^ Biochemistry and Molecular Genetics Department Hospital Clinic of Barcelona and Institut d'Investigacions Biomèdiques August Pi i Sunyer (IDIBAPS) Barcelona Spain; ^2^ CIBER of Rare Diseases (CIBERER) Instituto de Salud Carlos III Madrid Spain; ^3^ Department of Paediatrics Hospital Clinico Universitario de Valencia Valencia Spain; ^4^ Department of Neonatology BCNatal, Hospital Clinic Barcelona Barcelona Spain; ^5^ Neuromuscular Unit Hospital Sant Joan de Déu Barcelona Spain; ^6^ Applied Research in Neuromuscular Diseases Institut de Recerca Sant Joan de Déu Barcelona Spain; ^7^ Neuromuscular and Ataxias Research Group Health Research Institute Hospital La Fe (IIS La Fe) Valencia Spain; ^8^ Neuromuscular Diseases Unit, Neurology Department Hospital Universitari i Politècnic La Fe Valencia Spain; ^9^ Neuromuscular Reference Centre ERN‐EURO‐NMD Valencia Spain; ^10^ Department of Medicine University of Valencia Valencia Spain; ^11^ Laboratory of Neurogenetics and Molecular Medicine – IPER Institut de Recerca Sant Joan de Déu Barcelona Spain; ^12^ Department of Pathology Hospital Clinic Barcelona Spain; ^13^ Department of Pathology Hospital Sant Joan de Déu Barcelona Spain

**Keywords:** congenital muscular dystrophy, congenital myopathy, essential/alkali myosin light chain, fast‐twitch type II muscle fibres, muscle cell autophagy, myosin heavy chain

## Abstract

Congenital myopathies and congenital muscular dystrophies encompass heterogeneous clinical and genetic groups of disorders characterised by muscle weakness with antenatal or early postnatal onset. These conditions are categorised according to distinctive myopathological features and causative genes. Despite advances in diagnosis through massive parallel sequencing and progress in understanding the underlying pathogenesis, many aspects of these disorders remain poorly understood. *MYL1*‐related congenital myopathy is an ultra‐rare and severe condition, associated with a deficiency of essential/alkali light myosin and impaired development of fast‐twitch type II muscle fibres. This study aims to advance the understanding of the phenotype and pathogenesis of MYL1‐congenital myopathy.

We analysed the clinical characteristics of two individuals harbouring three novel variants in the *MYL1* gene. We conducted detailed genomic analysis and extensive studies on their muscles using histological, immunohistochemical, immunofluorescence, Western Blot and electron microscopy. Both individuals showed a very severe congenital myopathy, characterised by congenital hypotonia and weakness, requiring ventilatory and nutritional assistance. Muscle biopsy revealed dystrophic‐like or myopathic changes, with notable smallness of fast‐twitch type II fibres, often arranged around larger type I fibres, drawing a floret pattern. These fibres expressed developmental myosin and exhibited features of aberrant myofibrillogenesis. Type I myofibres exhibited correct sarcomere alignment, but like the small fast‐twitch fibres, both showed distorted cell organelles, vacuolar aggregates and membranous debris, indicating autophagic impairment.

Our findings confirm that bi‐allelic *MYL1* variants are associated with a severe congenital myopathy, characterised by a distinctive clinical and histopathological phenotype involving impaired type II fibre development. Additionally, our study reveals a role for MYL1 in the organisation and trophism of all muscle fibre types.

**Summary:**

- *MYL1* biallelic variants cause severe congenital myopathy with early hypotonia and type II fibre hypotrophy.
- Muscle biopsy shows a distinct pattern, including floret‐like fibre arrangement.
- Findings suggest a broader role for MYL1 in fibre organisation and autophagy across muscle fibre types.

## Graphical abstract

This study describes two individuals with severe congenital myopathy caused by novel biallelic *MYL1* variants. Detailed muscle analyses revealed selective type II fibre hypotrophy, aberrant myofibrillogenesis, and signs of autophagic impairment. The findings expand the clinical and pathological spectrum of MYL1‐related myopathy and suggest a broader role for *MYL1* in muscle fibre development and maintenance.

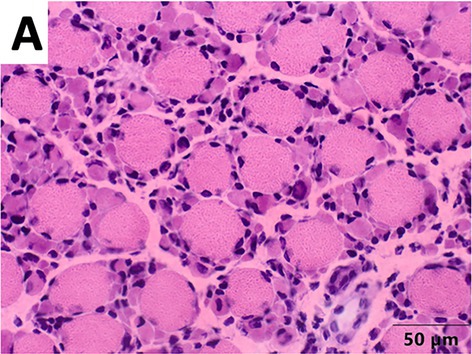

**Abbreviations**
CM: congenital myopathies
CMD: congenital muscular dystrophies
COX: cytochrome c oxidase
ELC: essential light chain
MLC: myosin light chain
NADH: reduced nicotinamide adenine dinucleotide tetrazolium reductase
RLC: regulatory light chain
SDH: succinate dehydrogenase

## 1 Introduction

Congenital myopathies (CM) and congenital muscular dystrophies (CMD) encompass heterogeneous groups of disorders characterised by early‐onset muscle weakness and distinct myopathological abnormalities [1, 2, 3]. CM are typically recognised by neonatal hypotonia, sometimes preceded by decreased intrauterine movements, and are classified according to specific muscle structural hallmarks [2, 4, 5]. Over 40 genes have been associated with CM [6] (http://musclegenetable.fr/, Jan 2024), coding for proteins mainly involved in the sarcomere assembly and the excitation‐contraction system [5, 7]. CMD are characterised by dystrophic muscle features, weakness, contractures and variable involvement of the respiratory, cardiac, central nervous and ocular systems [3]. The classification of CMDs is based on the type and location of the defective muscle proteins, with at least 35 causative genes implicated in processes such as extracellular matrix interactions, basal lamina integrity, glycosylation pathways, nuclear envelope maintenance and various organelle functions [3, 8]. Advances in the knowledge of these conditions are revealing significant overlap both within and between CM and CMD, challenging traditional pathological classifications. Distinct pathological profiles can be associated with different genes, and a single gene can manifest diverse phenotypes [5, 7, 8]. This overlap also extends to related disorders, such as congenital myasthenic syndromes [9] or arthrogryposis multiplex congenita [10]. Despite advances in genetic diagnostics, a high proportion of patients remain genetically undiagnosed [2].

The *MYL1* gene, which encodes a fast skeletal muscle‐specific essential light chain (ELC), has been shown to play an important role in myofibre development and function at the experimental level [11]. However, human disorders associated with *MYL1* remain poorly understood. To date, only two individuals have been reported with biallelic pathogenic *MYL1* variants, presenting with congenital myopathy and respiratory insufficiency (#618414; CMYP14) [12]. One of the propositi displayed dystrophic‐like muscle features and selective smallness of fast‐twitch type II fibres, while the other showed myopathic changes, type I fibres uniformity and nearly absent type II fibres. Questions lingered over whether the observed smallness or paucity of type II fibres stemmed from defective development or were secondary to a dystrophic process.

In this study, we present two unrelated individuals harbouring three novel recessively inherited loss‐of‐function *MYL1* variants, manifesting a severe congenital myopathy with prominent involvement of type II fibres, echoing the report by Ravenscroft et al. [12]. We unveil novel pathological features, shedding light on the crucial role of the *MYL1* gene in myofibrillar development and its contribution to the proper organisation of muscle cell organelles and cellular autophagy.

## 2 Methods

### 2.1 Recruitment of Individuals, Clinical Examination, Sample Collection and Processing

Data of individuals with *MYL1*‐related congenital myopathy were collected in accordance with the ethics guidelines of the Hospital Clinic of Barcelona and Hospital Clinico Universitario, Valencia. Written informed consent for study participation was obtained, including consent to publish clinical details, photographs and video material. Comprehensive clinical examinations were conducted on both individuals included in this study, and DNA analysis, electromyograms, muscle biopsies and autopsy studies were performed for diagnostic purposes. Myopathological studies for Individuals 1 and 2 were conducted at the Department of Pathology of Hospital Sant Joan de Déu, Barcelona and the neuromuscular research laboratories of IIS la Fe, Valencia, respectively.

### 2.2 Molecular Genetic Analyses

DNA was extracted from peripheral blood samples of the two unrelated probands and their unaffected parents using standard techniques. For Individual 1, an Illumina Nextera Flex for Enrichment exome capture was used to capture the coding regions on a NextSeq 500 sequencer (Illumina, San Diego, CA, USA), achieving a coverage of > 95% of targets with a minimum depth of 20× and a mean coverage of 140×. For Individual 2, the DNA sample was sequenced using SureSelectXT Human All Exon V6 (Agilent Technologies, Santa Clara, CA, USA) on an Illumina HiSeq sequencer (Illumina). Bioinformatic analysis involved aligning the short reads to the reference human genome (hg38) using BWA MEM (v0.7.17) and Bowtie2 (v2.4.1) short reads aligners, genotyping using Haplotype Caller from Genome Analysis Toolkit (v.4.2) and VarDict (v1.7.0) variant callers and annotation using Ensembl Variant Effect Predictor (v104). Variation annotation was performed using Annovar (v2018Apr16).

Variants were annotated using the reference sequences NM_079420.3 (transcript) and P05976 (protein). Validation and segregation studies of *MYL1* variants were conducted using Sanger sequencing.

### 2.3 *In Silico* Analyses of Novel *MYL1* Variants

*In silico* analysis of the genetic variants was performed using CADD [13], Mutation taster [14] and FATHMM‐MKL [15]. To evaluate splicing variants, we used Splice AI [16] and NNSplice (https://www.fruitfly.org/seq_tools/splice.html). We classified the variants following the guidelines of the American College of Medical Genetics and Genomics and the Association for Molecular Pathology (ACMG‐AMP) [17], using Franklin (https://franklin.genoox.com/clinical‐db/home). Additionally, previously reported variants were obtained from the Human Gene Mutation Database. To assess the affected residues and tolerance landscape, we used the MetaDome web server v.1.0.1 [18].

### 2.4 Muscle Biopsy and Autopsy Studies

We obtained muscle biopsy specimens from the quadriceps of Individual 1 and the anterior rectus abdominis of Individual 2. Additionally, post‐mortem muscle specimens from the deltoids, psoas and quadriceps of Individual 1 were collected. Transversally oriented muscle samples were frozen in isopentane chilled in liquid nitrogen and stored at −80°C until needed.

#### 2.4.1 Histological Studies

These specimens were processed using routine muscle histopathological techniques, including haematoxylin‐eosin, modified Gömöri's trichrome, reduced nicotinamide adenine dinucleotide tetrazolium reductase (NADH), succinate dehydrogenase (SDH), cytochrome c oxidase (COX), menadione‐linked glycerophosphate, periodic acid‐Schiff and Oil Red O stains [19].

#### 2.4.2 Immunohistochemical Studies

Muscle samples from Individual 1 were assessed for neonatal myosin heavy chain (NCL‐MHCn, Leica Biosystems), fast myosin heavy chain (NCL‐MHCf, Leica Biosystems) and slow myosin heavy chain (NCL‐MHCs, Leica Biosystems) expression using the Novolink Polymer Detection Systems kit (RE7290‐ce, Leica Biosystems).

#### 2.4.3 Immunofluorescence Studies

Immunofluorescence was performed on 8–10 μm‐thick muscle sections from both individuals. The sections were fixed with 4% paraformaldehyde for 7 min at room temperature, then rinsed with PBS‐Tween 0.5% and blocked for 90 min with PBS‐Tween 1% and BSA 8% at room temperature. Specific primary antibodies α‐MYL1 (1:20, PA5–29635, ThermoFisher), α‐laminin β1 (1:1000, MAB1921, Merck Millipore) and myosin heavy chain (NCL‐MHCn, Leica Biosystems) were incubated overnight at 4°C. After rising with PBS‐tween‐triton, secondary labelled antibodies α‐mouse Alexa Fluor 488 (1:500, A‐21202, ThermoFisher) and α‐rabbit Alexa Fluor 594 (1:500, A‐21207, ThermoFisher) were incubated for 3 h at room temperature in the dark. Sections were then rinsed and mounted with Fluoromount (00‐4958‐02, ThermoFisher). Immunofluorescence studies with primary antibodies against slow myosin A4.951 (sc‐53090), fast myosin‐2/MYH2 A4.74 and myosin heavy chain (NCL‐MHCd, Leica Biosystems) were performed in Individual 2.

Immunofluorescence images were acquired using a Leica microscope (Leica Microsystems) or an Olympus BX50 fluorescence microscope with a Nikon NIS‐Elements camera and analysed with ImageJ software.

#### 2.4.4 Western Blot Studies

Total protein from the muscle tissue of Individual 1 was extracted with RIPA lysis buffer (Bio Basic) containing a protease inhibitor cocktail (Bio Basic) for western blot studies. We homogenised the sample lysate using pellet pestles (Merck) and centrifuged it at 10,000 rpm for 5 min. We performed the western blot following conventional protocols. Precast gels TGX 4%–15% gradient (Bio‐Rad Laboratories) were used and transferred onto nitrocellulose membranes (Bio‐Rad Laboratories). Membrane blocking was performed using Odyssey Blocking Buffer (LI‐COR Biosciences). The membrane was incubated with primary antibody anti‐MYL1 (1:1000, PA5‐29635, ThermoFisher) and anti‐tubulin (1:1000, ab7291, Abcam) and with secondary antibodies IRDye 680LT goat anti‐rabbit (925‐68021, LI‐COR) and IRDye 800CW goat anti‐mouse (926‐32210, LI‐COR). Protein bands were visualised at the Odyssey LICOR fluorescent system (LI‐COR), and further analysis was performed using ImageJ software.

#### 2.4.5 Electron Microscopy

Electron microscopy was carried out on the muscle biopsies of Individuals 1 and 2. A standard protocol was applied to Individual 1's sample as previously described [20]. Frozen tissue samples from Individual 2 were quickly placed in ice‐cold Trump's fixative and maintained for 2 h, then transferred to a 4°C refrigerator and allowed to fix overnight; following fixation, the tissue was washed with phosphate buffer, post‐fixed in 1% osmium tetroxide and then processed accordingly to standards. Ultrathin sections were examined with a transmission electron microscope, JEOL model 1100 in Individual 1 and FEI Tecnai Spirit BT (ThermoFisher) in Individual 2. Electron micrographs were obtained using the Gatan Orius CCD camera (Olympus Soft Imaging Solutions, Münster, Germany).

## 3 Results

### 3.1 Clinical Features

**FIGURE 1:**
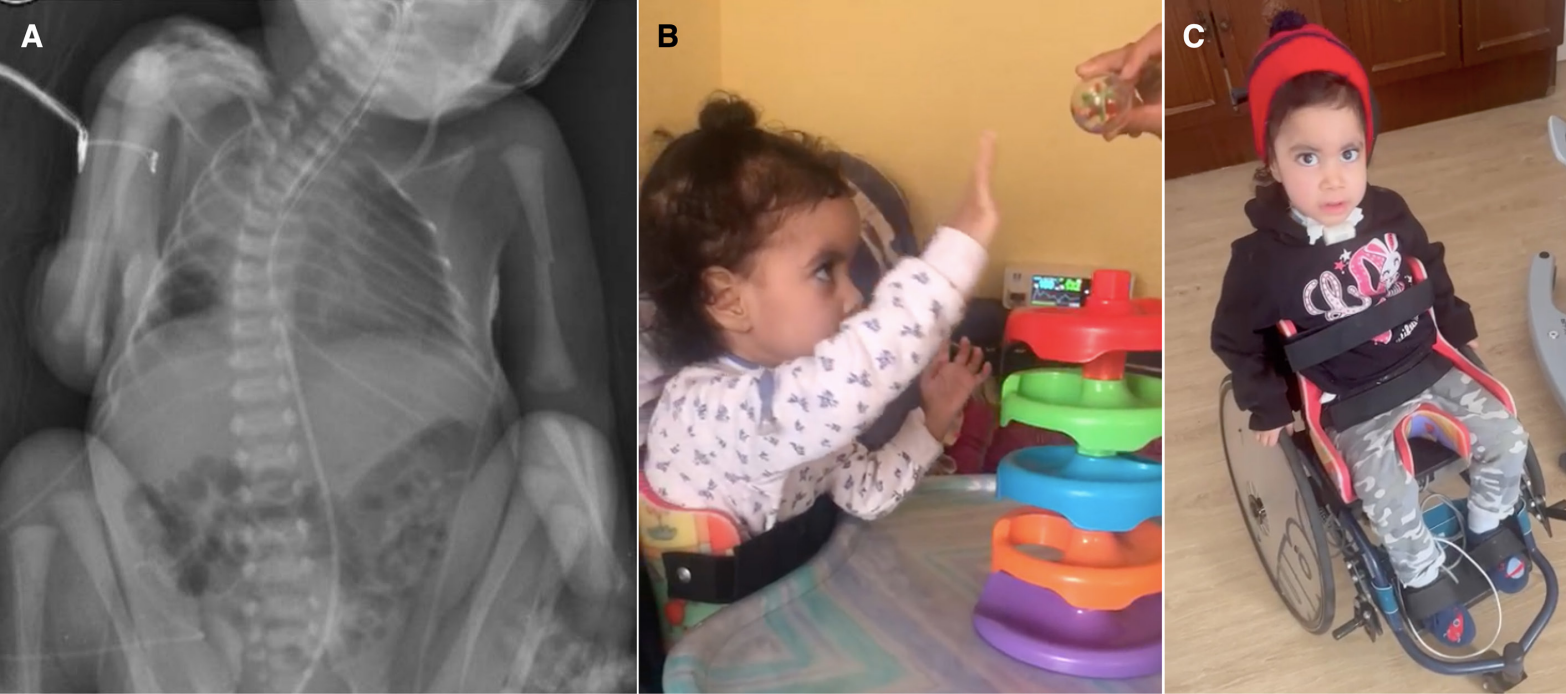
Clinical images of the two individuals with MYL1‐related congenital myopathy. (A) Thoraco‐abdominal radiograph of Individual 1 at birth, revealing a bilateral humerus fracture. The fracture is displaced on the left side and nondisplaced on the right side. (B) Lateral photograph of Individual 2 at 2 years and 4 months, demonstrating head control and the ability to elevate upper limbs against gravity. (C) Frontal photograph of Individual 2 at 2 years and 9 months, highlighting preserved strength in the upper limbs and hands compared to the lower limbs, as she is even able to propel her wheelchair.

Table 1 provides a summary of the clinical features of Individuals 1 and 2 and those of the two previously reported individuals with MYL1‐related congenital myopathy. Individual 1 was the first child of non‐consanguineous Caucasian parents with no family history of neuromuscular disease. The pregnancy was complicated by severe polyhydramnios, fetal growth restriction and reduced fetal movements, leading to an emergency caesarean section at 34.5 weeks of pregnancy. At birth, the newborn weighed 1640 g (p3; −1.87 SD), had a crown‐heel length of 45 cm (p35; −0.38 SD) and a head circumference of 32 cm (p54; + 0.10 SD). The Apgar scores were 2 at minute one and 5 at 5 min, indicating poor respiratory effort. He required the placement of an endotracheal tube ventilation in the delivery room due to respiratory insufficiency. Physical examination during the neonatal period revealed severe generalised weakness, including facial weakness with a high‐arched palate, retrognathia and wide philtrum, generalised areflexia, elongated phalanges, thoracic scoliosis, bilateral cryptorchidism, long and thin bones and a fracture in the humerus that occurred during delivery (Figure 1A). Arthrogryposis, ptosis and ophthalmoplegia were not observed. Since he had severe dysphagia and marked drooling, nasogastric feeding was required. Creatine kinase (CK), ammonium, echocardiogram, funduscopic examination and brain and abdominal ultrasounds were normal. Genetic testing for spinal muscular atrophy (SMA), Prader‐Willi syndrome and myotonic dystrophy Type 1 was also normal. One week after birth, he developed a right pneumothorax, and 10 days after birth, a necrotising pneumonia caused by *Staphylococcus aureus* complicated by a right pleural effusion requiring drainage. Secondary to generalised infection, he developed renal failure, hypoalbuminaemia and anasarca and eventually died of respiratory failure at 1 month of age.

**TABLE 1:** Clinical features of individuals with *MYL1* gene variants. F female, M male, CPAP Continuous positive airway pressure.

	Individual 1 present work	Individual 2 present work	Individual 1 in Ravenscroft et al.	Individual 2 in Ravenscroft et al.
Sex	M	F	M	F
Variants	c.334C > T (p.Gln112Ter) c.478 + 1G > A	c.543del (p.Cys181Ter) c.543del (p.Cys181Ter)	c.479‐2A > G c.479‐2A > G	c.488 T > G p.(Met163Arg) c.488 T > G p.(Met163Arg)
Age at last examination	1 month (deceased)	2 years	7 months (deceased)	3 years
Gestational age at birth (in weeks)	34	38	36	37
Prenatal findings	Polyhydramnios, fetal growth restriction, reduced intrauterine movements	Polyhydramnios, reduced intrauterine movements	Polyhydramnios	Reduced intrauterine movements
Neonatal period	Poor respiratory effort requiring ventilation via endotracheal tube	Poor respiratory effort requiring ventilation via endotracheal tube	Poor respiratory effort requiring ventilation via endotracheal tube	Poor respiratory effort requiring ventilation via endotracheal tube
Respiratory involvement	Ventilation via endotracheal tube	Ventilation via endotracheal tube and subsequent tracheostomy (currently intermittent positive pressure via tracheostomy)	Ventilation via endotracheal tube (first 3 months of life) and subsequent CPAP.	Ventilation via endotracheal tube and subsequent tracheostomy (currently nocturnal positive pressure via tracheostomy)
Feeding	Severe dysphagia (nasogastric tube required for feeding)	Severe dysphagia (enteral nutrition by gastrostomy tube)	Severe dysphagia (nasogastric and gastrostomy tubes required for feeding)	No feeding difficulties
Maximal motor ability	Died at the age of 1 month	Head control and antigravity movements in the upper limbs	Some partial antigravity movements in the upper and lower limbs	Sit unaided at 24 months and walk with support at 3 years of age.
Bone fractures	Humerus fracture during delivery	Metaphyseal fracture of the distal right femur at 2 years old	Not reported	Not reported

Individual 2 was a female who was the third child of consanguineous parents of Moroccan origin, with no reported family history of neuromuscular disease. Her mother had four term deliveries, including two stillbirths and two live births, as well as one miscarriage. No genetic studies or reports were available regarding the cause of the stillbirths or the miscarriage. The pregnancy was complicated by polyhydramnios and reduced fetal movements and the individual was born by caesarean section at 38 + 3 weeks gestation with a weight of 2545 g (p8; −1.41 SD). Apgar scores at minutes one and five were 6 and 8, respectively. The newborn was floppy, and the respiratory effort was weak, requiring endotracheal tube ventilation. Physical examination in the neonatal period showed severe generalised weakness, including facial weakness, decreased eye‐opening, hypertelorism, wide nasal dorsum, prominent forehead and low‐set ears, as well as joint hypermobility and decreased spontaneous movements, without joint contractures. Deep tendon reflexes were absent and further observations included clasped thumbs, a marked medial plantar arch and a bell‐shaped chest. Cardiac examination and electrocardiography were normal and a patent foramen ovale of minimal size was observed in the echocardiogram. Brain ultrasound and brain MRI showed increased extra‐axial cerebrospinal fluid spaces compatible with benign external hydrocephalus and EEG and CSF analysis were normal. CKs were normal. Nerve conduction studies and repetitive nerve stimulation at 3 Hz were normal; low‐amplitude and short‐duration motor unit potentials, consistent with a myopathy, were found in the electromyography. Single‐fibre electromyography was not performed. Genetic testing for SMA and myotonic dystrophy Type 1 was normal.

A gastrostomy tube was placed at 5 months of age due to persistent weak suction and is currently maintained with total enteral nutrition. By the age of 6 months, multiple attempts to discontinue invasive mechanical ventilation were unsuccessful, leading to the placement of a tracheostomy for intermittent respiratory support with BiPAP. Increasingly longer disconnections from respiratory support have been possible over time, progressing from approximately 20 min twice a day to 4–6 h since the age of two and this remains the case at 3 years and 10 months old. Her clinical condition has shown gradual improvement over the first years of life. At the latest examination at age 3 years 10 months old, she presented with generalised weakness and could control her head but was unable to sit without support or to walk (Figure 1B,C). However, she was able to handle light objects with both hands and point with her fingers (Video S1). Like Individual 1, Individual 2 has also experienced bone fractures, specifically a metaphyseal fracture of the distal right femur at 2 years of age, which recurred at 3 years in the same location after slight movement.

### 3.2 Molecular Genetics

**FIGURE 2:**
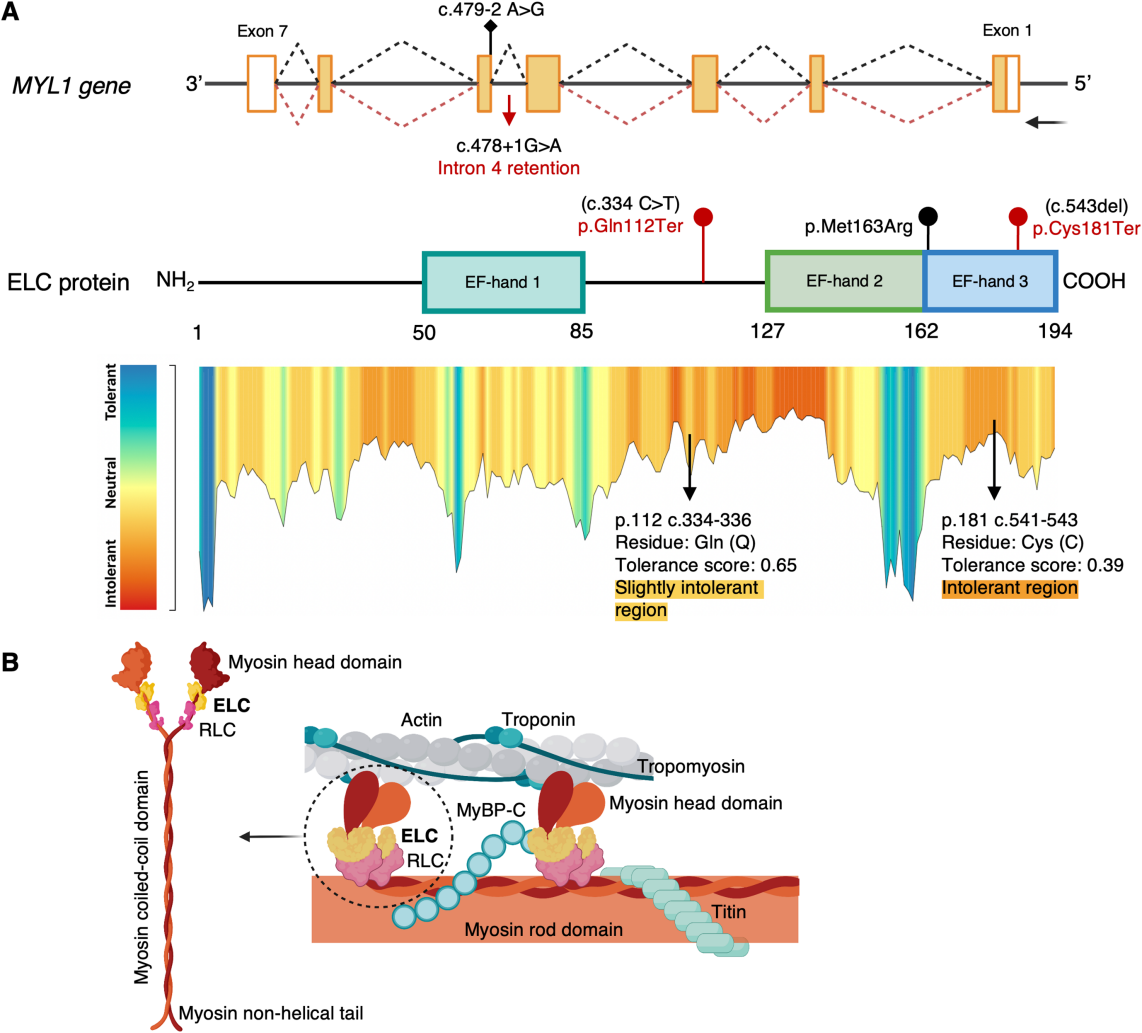
*In silico* evaluation of *MYL1* genetic variants found in Individuals 1 and 2. (A) The upper panel shows the *MYL1* gene structure, which codifies the skeletal muscle fast‐twitch specific myosin essential light chain (ELC). ELC domain structure (Uniprot: P05976) is represented in the middle panel. Genetic variants found in Individuals 1 and 2 are indicated with red symbols, and previously reported variants are represented in black (in the upper panel if they are splicing variants and in the middle panel those that are missense or truncating). Previously reported variants (according to the Human Genome Mutation Database) are associated with severe congenital myopathy. The lower panel shows the tolerance landscape of ELC according to the MetaDome web server, indicating the positions of the truncating variants relative to regions of varying tolerance to variation. (B) Schematic drawing of protein interactions in striated muscle, highlighting the major myosin regulatory proteins. Abbreviations: ELC: myosin essential light chain; RLC: myosin regulatory light chain‐2; MyBP‐C: myosin‐binding protein.

Exome Sequencing identified the two compound heterozygous *MYL1* (NM_079420.3) variants c.334C > T (p.Gln112Ter) and c.478 + 1G > A in Individual 1 and a homozygous *MYL1* variant c.543del (p.Cys181Ter) in Individual 2 (Figure 2). The three variants are classified as likely pathogenic according to the American College of Medical Genetics and Genomics (ACMG) guidelines (last accessed June 2024) [17]. These variants were absent from the gnomAD database, except for c.478 + 1G > A, which had a very low allelic frequency of < 0.0001. The *in silico* predictors supported the pathogenicity of these variants, with c.334C > T (p.Gln112Ter) and c.543del (p.Cys181Ter) variants leading to premature stop codons and having CADD scores of 38 and 33, respectively. The *MYL1* c.478 + 1G > A variant is located at a canonical splice‐donor site (Exon 4—Intron 4), is predicted to affect splicing according to Splice AI v1.3.1 [16] and has a CADD score of 34 (Table S1). According to *in silico* predictions, the loss of the canonical donor site (SpliceAI score: 1.0; position: 1 bp) and the potential activation of an alternative site within Intron 4 (SpliceAI score: 0.20; position: 34 bp) would result in the inclusion of part of Intron 4 in the transcribed mRNA.

Sanger sequencing analysis of the unaffected progenitors of both index individuals revealed that each parent carried one of the variants in heterozygous form. Specifically, the father of Individual 1 carried the *MYL1*: c.334C > T variant, the mother of Individual 1 carried the *MYL1*: c.478 + 1G > A variant and both parents of Individual 2 carried the *MYL1*: c.543del variant (Figure S1). No additional variants of interest, defined as variants classified as pathogenic in ClinVar, in relevant disease‐causing genes were found.

### 3.3 Pathological Findings in Muscle Biopsies

#### 3.3.1 Histology and Immunohistochemical Analysis

##### 3.3.1.1 Individual 1

**FIGURE 3:**
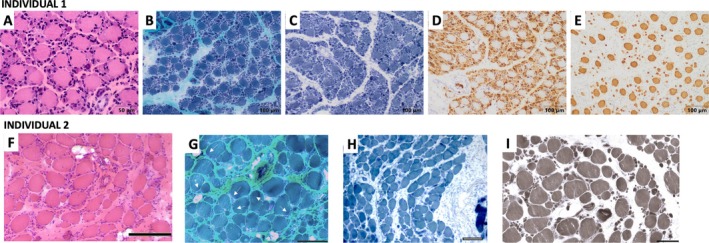
Histopathological findings in the muscle samples of individuals with *MYL1*‐related congenital myopathy. Upper row pictures correspond to Propositus 1 and lower row pictures to Propositus 2. Haematoxylin‐eosin in Individuals 1 (A) and 2 (F) shows marked variability in the size of muscle fibres, with a population of hypertrophic fibres surrounded by small fibres. Fibrosis and fatty infiltration were more prominent in Individual 2, as observed in (F). Modified Gömöri trichrome staining in Individuals 1 (B) and 2 (G) shows an increase in endomysial and perimysial connective tissue. Dark purple granular inclusions (arrows) were identified in Individual 2 (G). SDH in Individual 1 (C) and NADH‐TR histochemistry in Individual 2 (H) showed uniform staining in larger fibres and a mixed pattern of dark and light in the smaller fibres. Fast myosin immunohistochemical staining of Individual 1 (D) showed that small fibres were fast fibres, and slow myosin immunohistochemical staining showed that all hypertrophic fibres of Individual 1 (E) were slow fibres. Similarly, ATPase 9.4 (I) and ATPase 4.6 (not shown) in Individual 2 showed that all small fibres corresponded to IIA or IIC with no presence of IIB/IIX, whereas the large fibres were all type I.

Muscle tissue was obtained from the quadriceps at 19 days of age. Histological examination revealed marked myopathic changes, including high variability in fibre size (2–50 μm; normal for age 10–15 μm), internalised nuclei and minimal perimysial and endomysial fibrosis, as observed with haematoxylin‐eosin staining (Figure 3A). A peculiar pattern was noted, with large fibres surrounded by small fibres, resembling small flowers (floret‐like). Modified Gömöri trichrome staining did not reveal any visible inclusions (Figure 3B). SDH staining showed uniform reactivity in larger fibres, while some smaller fibres exhibited increased reactivity, giving them a darker appearance (Figure 3C). Most small fibres co‐expressed fast and neonatal myosin and were situated around larger fibres that expressed only slow myosin, creating a distinct ‘floret’ appearance as described by Ravenscroft et al. [12] (Figure 3D). PAS and Oil red O staining showed no abnormalities (not shown). Isolated small fibres showed positive immunostaining for both slow and fast myosin, indicating co‐expression (Figure 3E).

##### 3.3.1.2 Individual 2

Conventional preparations of the muscle sample taken at 5 months of age displayed pronounced disruptions in muscle fascicular architecture, including perimysial and endomysial fibrosis and fatty infiltration. A significant variation in myofibre size was observed (3–90 μm; normal range for age: 15–20 μm). The smaller fibres appeared scattered or clustered into small groups across the endomysium or surrounding a large myofibre in a ‘floret’ pattern (Figure 3F,G). ATPase histochemistry showed that larger fibres were uniformly type I, while small or very small fibres reacted as fast‐twitch IIA or C (Figure 3I). Apart from occasional nuclear centralisation and splitting sarcolemmal infoldings, the most relevant structural abnormalities included basophilic granulation in small fibres and dark purple granular inclusions seen on Gömöri staining (Figure 3G), which reacted negatively for menadione‐linked glycerophosphate. NADH‐TR reaction showed uniform staining in larger fibres and a mixed pattern of dark and light in the smaller fibres (Figure 3H). No active necrosis, myophagia or inflammatory cell infiltration was observed.

#### 3.3.2 Immunofluorescence Analysis

**FIGURE 4:**
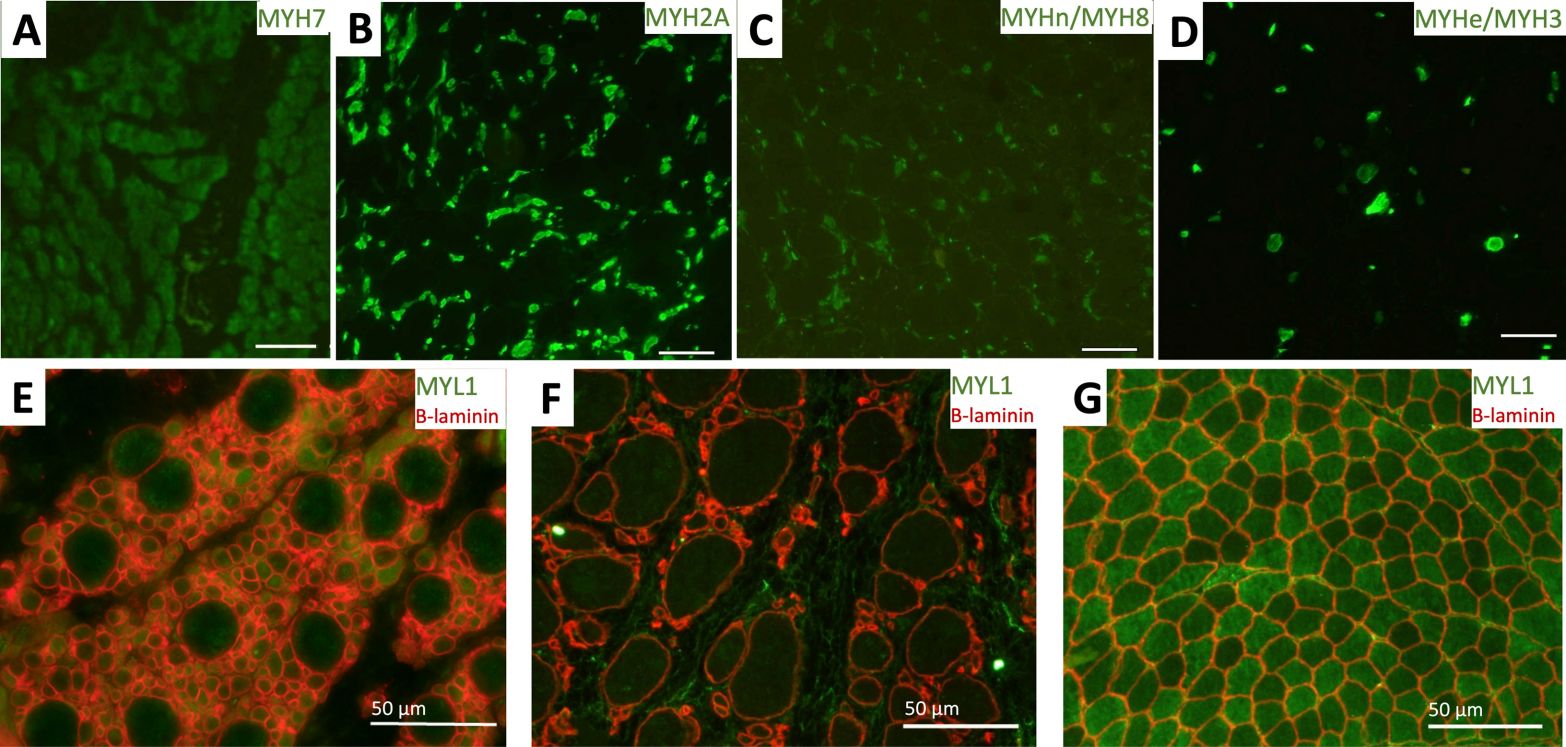
Immunofluorescence assays on muscle samples from individuals with *MYL1*‐related congenital myopathy The upper row (A‑D) shows immunofluorescence images showing the expression of slow myosin (MYH7) (A), fast myosin (MYH2A) (B), embryonic myosin (MYH3/MYHe) (C) and neonatal myosin (MYH8/MYHn) (D) in Individual 2. Note that MYH7 is mainly expressed in large fibres and a few scattered small fibres (A). MYH2A expression is restricted to small fibres (B), with a high proportion of these small fibres co‐expressing MYH8 (C), while only a small proportion of them also co‐expressed MYH3 (D). The lower row presents double immunofluorescence staining of MYL1 (green) and b‐laminin (red) in Individual 1 (E) and Individual 2 (F), compared to control muscles (G). In Individual 1 (E), a reduction in MYL1 expression is observed, while in Individual 2 (F), MYL1 expression is absent. In contrast, the control muscle (G) showed two intensities of immunostaining with a checkerboard distribution, with low or very low expression in type I fibres.

Immunofluorescence assays revealed a distinct pattern of myosin expression. Larger fibres, along with a few scattered small fibres, expressed slow myosin (Figure 4A), whereas smaller fibres predominantly expressed fast myosin (Figure 4B). A notable proportion of these smaller fibres also co‐expressed neonatal myosin (Figure 4C), and a few small fibres co‐expressed embryonic myosin as well (Figure 4D). In Individual 1, immunofluorescence staining for MYL1 revealed a proportion of fibres with reduced fluorescence intensity, with some fibres completely lacking MYL1 expression (Figure 4E). In contrast, Individual 2 showed a complete absence of MYL1 expression in all myofibres (Figure 4F). Muscle control samples displayed the typical checkerboard pattern, with high MYL1 expression in type II fibres and low or very low intensity in type I fibres (Figure 4G). Immunolabelling of sarcolemmal proteins showed normal staining (not shown).

### 3.4 Western Blot Findings

Muscle extracts from the quadriceps sample of Individual 1, as well as from the deltoid, psoas and quadriceps samples obtained postmortem, showed a lower intensity of MYL1 protein compared to the control muscles. The amount of MYL1 protein was lower in the psoas and quadriceps muscles compared to the amount in the deltoids (Figure S2).

### 3.5 Ultrastructural Study

**FIGURE 5:**
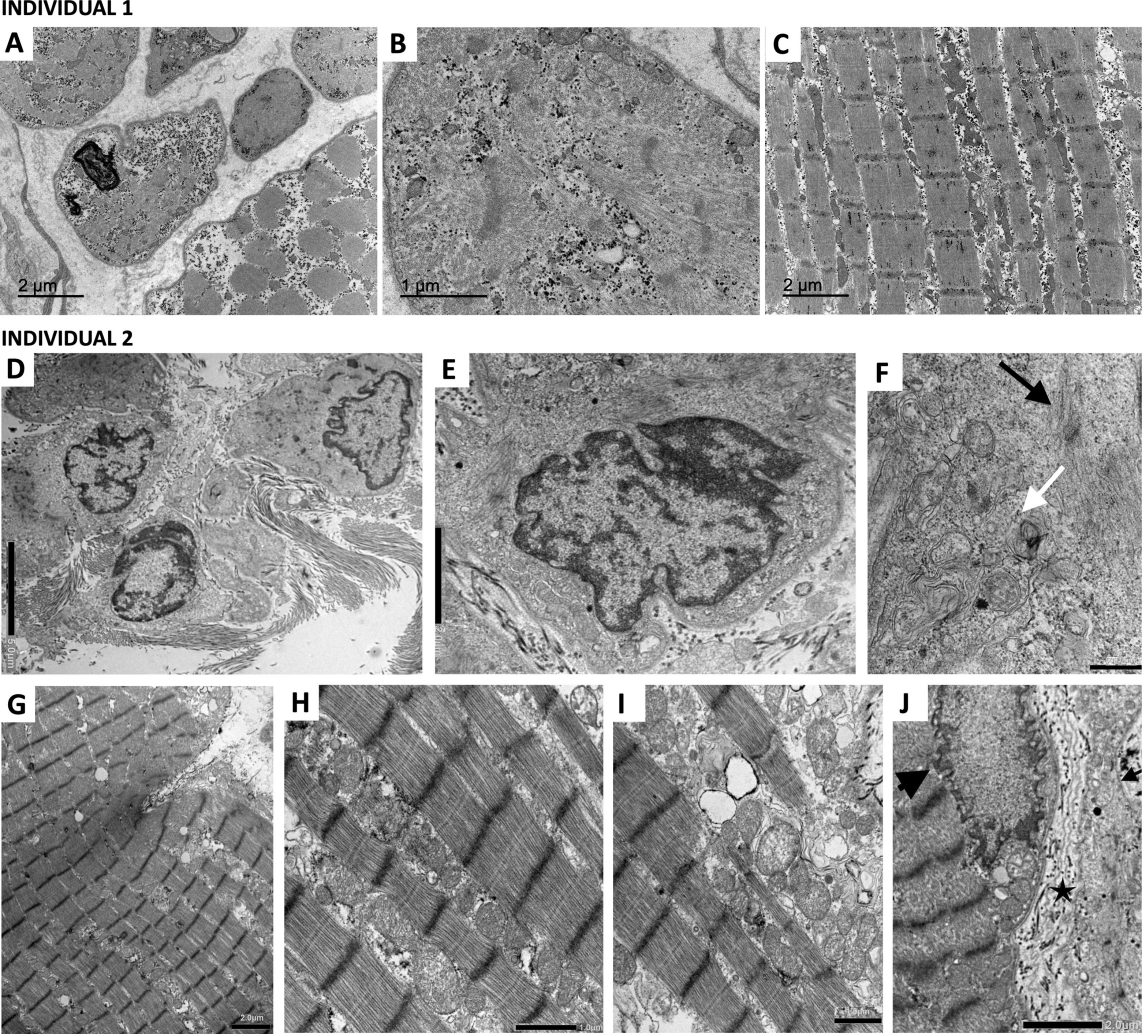
Electron microscopy of muscle samples from individuals with *MYL1*‐related congenital myopathy. The upper row (A‑C) shows electron micrographs of muscle samples from Individual 1. Small fibres (type II) show a range of myofibrillar abnormalities, with diversity in frequency and size, as well as disrupted sarcomeric organisation (A, B). The sarcoplasm of these small fibres contained areas of electron‐dense aggregates whose subcellular structure is indistinct. Some small fibres also showed centralised nuclei, without evidence of redundant basement membrane folding. Micrograph (C) shows a large fibre (type I) with well‐preserved myofibrils and aligned sarcomeric Z‐bands. Additionally, intermyofibrillar aggregates are visible, including elements with the shape of mitochondria. The middle and lower rows (D‑J) show electron micrographs from Individual 2. Micrograph (D) shows a collection of small muscle fibres with immature features embedded in a fibrous background. Micrographs (E) and (F) provide detailed views of a small muscle fibre, where the sarcoplasm contains sparse filamentous bundles attached to miniature Z‐disks, suggestive of myofibril precursors (**black arrow**). Additionally, membranous aggregates are visible, including vacuoles, complex membrane folding and myelin‐like features (**white arrow**). Micrograph (G) corresponds to a large type I myofibre with an invagination of the sarcolemma, suggestive of incipient fibre splitting. The fibre has well‐preserved myofilaments, proper sarcomere organisation and aligned Z bands. This large fibre depicts aggregates of vacuoles, degraded mitochondria and membranous structures of diverse sizes within the intermyofibrillar (H) and subsarcolemmal (I) spaces. In micrograph (J), proliferation of basal lamina folds (**star**) appears in the extracellular space spanning between a large type I fibre (**large black arrow**) and adjacent small myofibres with immature features (**small black arrow**). Finally, note the nuclear abnormalities in both small and large fibres, consisting of irregular shapes with infoldings and segmentations, abnormal chromatin distribution and nuclear envelope abnormalities.

Ultrastructural evaluation of muscle samples from Individuals 1 and 2 at low magnification confirmed the profile of small fibres arranged in groups, embedded within fibrous stroma or surrounding larger fibres in floret formations (Figure 5A,E). The small myofibres exhibited a wide range of myofibrillar abnormalities. In Individual 1, some fibres contained apparently mature but randomly oriented myofibrils with thick Z lines (Figure 5A,B), whereas in Individual 2, the small myofibres often showed sparse, thin fibril bundles attached to rudimentary Z‐disks, reminiscent of immature sarcomeres arrested at pre‐or‐nascent stages of myofibrillogenesis (Figure 5E,F). The sarcoplasm of these small fibres contained abundant areas of electron‐dense aggregates. In Individual 1, their structure was not discernible, while in Individual 2, these aggregates were recognisable as vacuoles and membranous arrangements with complex membrane folds and myelin‐like structures (Figure 5E,F).

Larger fibres (type I) showed normal myofibrillar organisation with preserved sarcomere registers. However, they also displayed profuse aggregates in both intermyofibrillar and subsarcolemmal spaces, consisting of collections of distorted mitochondria recognisable by their double membrane and cristae‐like profiles, as well as vacuoles and membranous structures similar to those observed in the small myofibres (Figure 5C, H–J). Occasional areas of basal membrane replication were observed in the extracellular space spanning between the sarcolemma of some large myofibres and their adjacent small immature myofibres (Figure 5K). Lastly, nuclear abnormalities, including infoldings, segmentation, uneven chromatin distribution and protrusions of the nuclear envelope, along with intermembrane space widening, were observed in both type I and type II myofibres (Figure 5A,D,E,J).

## 4 Discussion

In this study, we present two individuals from unrelated families with a severe congenital myopathy associated with biallelic variants in the *MYL1* gene. These variants lead to deficient expression of the skeletal muscle fast‐twitch specific myosin essential light chain (ELC). Our findings expand the understanding of *MYL1*‐related congenital myopathy, a condition previously reported only in two individuals of Turkish origin [12].

### 4.1 Myosin Essential Light Chain (ELC) in the Context of Myosinopathies

The landscape of diseases associated with the myosin ELC protein is not yet fully understood. These disorders can be classified within the broad category of myosinopathies, which include a large group of CM, arthrogryposis syndromes, early‐ and late‐onset cardiomyopathies and various myopathies with ocular, proximal or distal involvement [21, 22, 23, 24, 25, 26]. Such complexity of disorders is a consequence of the diverse roles played by the myosin II molecule in myogenesis, muscle regeneration, cytoskeleton organisation and cell signalling pathways, in addition to its essential function in muscle contraction [26, 27, 28, 29].

The myosin II molecule is a hexameric complex composed of two myosin heavy chains (MHCs) forming a dimer with a coiled‐coil tail and two heads housing the motor and catalytic domains. Each of the MHC flexible ‘neck’ regions includes IQ domains that bind the two myosin light chains (MLCs): the essential/alkali light chains (ELC) and the regulatory light chains (RLC) (Figure 2) [26, 30]. These light chains belong to the EF‐hand calcium‐binding protein family and play crucial roles in modulating the actin‐myosin cross‐bridge cycle [11].

### 4.2 Myosin Isoforms Regulate Muscle Diversity

Different isoforms of myosin heavy chains are encoded by distinct genes that dictate the physiological properties of body muscles [29]. In humans, adult skeletal muscles contain slow type I myosin (*MYH7*) and fast myosins, including type IIa (*MYH2*) and IIx (*MYH4*). Rodents also express type IIb myosin, which is absent in humans. During development, embryonic (*MYH3*) and neonatal (*MYH8)* myosin isoforms are highly expressed, but they are downregulated after birth, only to be re‐expressed during muscle regeneration [30]. The expression of the isotypes of myosin is canonically programmed at different stages of myogenesis; however, after birth, diverse factors like thyroid hormone, innervation and exercise promote the switch to adult‐specific isoforms, leading to different muscle allotypes based on the muscle functionality [31].

Similarly, the essential/alkali light chain presents five isoforms coded by four genes (*MYL1*, *MYL3*, *MYL4*, *MYL6B*), with *MYL1* encoding two spliced isoforms (MLC1f and MLC2f) [29]. On the other hand, two regulatory light chain isoforms are encoded by the *MYL2* and *MYLPF* genes [29]. Myosin light chain isoforms also possess myofibre specificity: *MYL1* is mainly involved in fast‐twitch muscle fibres (Type 2), whereas *MYL2* and *MYL3* are implicated in slow type I fibres and cardiac ventricles [12].

### 4.3 Diseases Associated With Myosin Light Chains

Pathogenic variants in *MYL2*, *MYL3* and *MYL4* genes cause various isolated cardiomyopathies [11, 32, 33, 34]. However, skeletal myopathies have only been associated with *MYL1* and *MYL2* genes [12, 33, 35]. This work focuses on *MYL1* while noting that biallelic *MYL2* pathogenic variants cause a myopathy with early onset after birth, leading to progressive cardiomyopathy and early death in infants (OMIM #619424) [33]. Pathologically, this condition is marked by type I fibre hypotrophy and myofibrillar disarray.

### 4.4 Clinical Profile of MYL1‐Related Congenital Myopathy and Clinico‐Genetic Correlations

The analysis of the phenotypes of the two individuals in our study and the two propositi previously reported with *MYL1*‐related congenital myopathy reveals a consistent congenital presentation characterised by severe generalised weakness, including involvement of respiratory and bulbar muscles, without cardiomyopathy (Table 1). The spectrum of causative variants leading to *MYL1* loss‐of‐function is broad, as observed in Table 1; however, the current number of reported cases remains too limited to establish a robust genotype–phenotype correlation. Nevertheless, it is noteworthy that Individual 1 in our study and Individual 1 described by Ravenscroft et al.'s report, both of whom experienced early fatal outcomes, carried splicing and frameshift variants in the central region of the gene, affecting the translation of EF‐hand Domains 2 and 3. In contrast, Individuals 2 in both studies, who reached longer survival, harboured nonsense or missense variants in the terminal region of the gene, affecting only the EF‐hand Domain 3. This observation may suggest a higher functional importance of EF‐hand Domain 2.

A discrepancy between MYL1 protein expression and clinical severity was observed in our two reported individuals. Individual 2, who showed a complete absence of MYL1 expression, experienced significant improvements in respiratory and motor functions during childhood. In contrast, Individual 1, who retained partial MYL1 expression, had a premature fatal outcome. A partial compensatory effect by other myosin isoforms could account for the progressive improvement observed in some patients, possibly contributing to longer survival. This hypothesis aligns with findings in large eutherian mammals, which shift toward a predominance of slow myosin isoforms in proportion to increasing body mass [36]. Similar late‐onset improvements attributed to compensation by related isoforms or paralogous genes have been described in CM associated with TNNC2 and HACD1 [37, 38].

A notable feature of *MYL1* congenital myopathy is the paradoxical preservation of strength in proximal upper limb muscles, rich in fast‐twitch fibres, compared to the poorer performance of lower limbs and axial muscles, which predominantly consist of slow‐twitch type I fibres. This suggests that MYL deficiency impacts both fast and slow twitch fibre types.

The involvement of respiratory and bulbar muscles may partially justify the rarity of this condition with implicit neonatal lethality. This fact could be related to a lack of response to the thyroid surge that arises in neonate mammals to promote the transition from developmental myosin isoforms toward MyH‐IIB/IIX, endowed with a faster rate of contraction to subserve the functional demand of laryngeal muscles at this stage [36]. The persistence of developmental myosins in MYL1 myopathy supports this speculation.

### 4.5 The Myopathological Profile of MYL1 Congenital Myopathy

The myopathological profiles of the individuals in our study are consistent with those described in Individual 1 of Ravenscroft et al. [12], characterised by a myopathic background with increased connective and adipose tissue and a proliferation of small fast‐twitch type‐II fibres. Whereas the atrophy or hypotrophy of type I fibres is a common feature in CM [2], the marked smallness of type II fibres observed in *MYL1*‐congenital myopathy rarely occurs in genetic myopathies, specifically in MYH2‐related ones [25, 35, 39, 40]. Interestingly, MYH*2* myopathies can associate a wide range of structural alterations [41] or manifest only mild myopathic changes with a particular profile of fibre type I uniformity [25] like the Individual 2 described by Ravenscroft et al. [12]. Selective type II fibre atrophy has also been observed as an associated feature in congenital myasthenic syndromes, steroid‐induced myopathies, critical illness myopathy and sarcopenia [12].

### 4.6 The Floret Appearance in MYL1 Congenital Myopathy

The distinctive presence of small, immature type II fibres surrounding larger type I fibres, creating a ‘floret’ appearance in *MYL1*‐related congenital myopathy, had not been previously described until Ravenscroft et al. [12]. The significance of this feature remains elusive, although it may indicate a developmental arrest at the myotube fusion stage or represent an abortive regenerative attempt by activated nearby satellite cells. The sparse proliferation of basal lamina in the scene is compatible with a possible degeneration/regeneration cycle. However, the surrounding ring of hypotrophic myofibres, decorated with immature features and retaining developmental myosin isoforms, points to a disruption in normal myogenesis.

### 4.7 Novel Pathological Features in MYL1 Congenital Myopathy

Muscle biopsies from the individuals described here reveal novel pathological features. On one side, the small fast‐twitch fibres exhibit myofibrillar abnormalities, including disordered assembly with sarcomeric disarrangements, suggesting profiles indicative of developmental arrest during early myofibrillogenesis [42]. This finding aligns with the fact that MYL1 is the only MLC isoform expressed in fast‐twitch fibres during early myogenesis [43]. It is also supported by studies involving *MYL1* knockdown models in myoblasts of chicken [44] and zebrafish [12]. In contrast, the preservation of sarcomeric structure in type I fibres correlates with the fact that MYL3 is the essential light chain isoform selectively present in slow fibres [45], potentially assuming the myofibrillogenesis role typically played by MYL1 in fast‐twitch fibres.

Another relevant observation in MYL1 pathology is the presence of abnormal conformational features across diverse muscle organelles, alongside profuse aggregates of vacuoles and membrane debris. The underlying cause of these abnormalities remains elusive; however, they unequivocally indicate that MYL1 plays a significant role in regulating organelle disposition and autophagic processes. Notably, these abnormalities are present in both fast‐ and slow‐twitch fibres, indicating that MYL1 contribute significantly to the trophism of type I fibres, in addition to its predominant expression in fast‐twitch fibres [45]. Intriguingly, nuclear abnormalities similar to those here described have been reported in other CM with variants in sarcomeric genes, having been analysed in detail in a transgenic model of nemaline myopathy with an *ACTN1* variant [46]. Remarkably, when this knocking mouse model was transfected with a transgene of the essential light myosin isoform MYL4, closely related to MYL1, the experimental animal demonstrated significant functional and pathological recovery.

## 5 Conclusions

Our study confirms that biallelic loss‐of‐function variants in *MYL1* are associated with a consistent clinical and histopathological phenotype, which should be considered in individuals with severe congenital myopathy, particularly those with selective hypotrophy of type II fibres. We reveal novel pathological features, shedding light on the crucial role of the *MYL1* gene in the development of type II myofibres, as well as its key contributions to the organisation of cell organelles and regulation of cell autophagy across all fibre types. Muscle biopsy is recommended in cases of severe congenital myopathy, especially when the genetic diagnosis is unclear, as it can reveal a distinctive myopathological pattern, as the one observed here in association with *MYL1* variants.

## Author Contributions

I.M., C.V.V., G.A., J.J.V. and D.N‐d.B. had a major role in the acquisition, analysis and interpretation of the data. I.M., C.V.V., J.J.V. and D.N‐d.B. drafted the manuscript for intellectual content. C.V.V. and D.N‐d.B. prepared Figure 1. B.E.A. prepared Figure 2. C.J., A.C., J.J.V. and D.N‐d.B. prepared Figures 3‑5. D.N‐d.B. is responsible for the overall content as the guarantor. G.A., J.E.E., L.R.R., A.P.L., N.M., R.V., M.C.C., L.C.G., C.O., L.R.C., G.S. and I.A. contributed to data collection, performed analyses and reviewed the manuscript for intellectual content.

## Ethics Statement

Data of individuals with MYL1‐related congenital myopathy were collected in accordance with the ethics guidelines of Hospital Clinico Universitario, Valencia, Hospital Clinic of Barcelona and Hospital Sant Joan de Déu (protocol PIC‐147‐23). Written informed consent for study participation was obtained.

## Consent

Written informed consent for publication was obtained.

## Conflicts of Interest

The authors declare no conflicts of interest.

## Supporting information


**Figure S1** Supporting information.

**Figure S2** Supporting information.

**Table S1** Pathogenic effect of *MYL1* variants found in Individuals 1 and 2.

**Video S1** Supporting information.

## Data Availability

The data that support the findings of this study are available on request from the corresponding author. The data are not publicly available due to privacy or ethical restrictions.
