# Supplementary figures and images for: MYL1‐Related Congenital Myopathy: Clinical, Genetic and Pathological Insights
Source: Neuropathol Appl Neurobiol. 2025 Jun 9;51(3):e70025. doi: 10.1111/nan.70025 (PMC12147433; doi:10.1111/nan.70025)

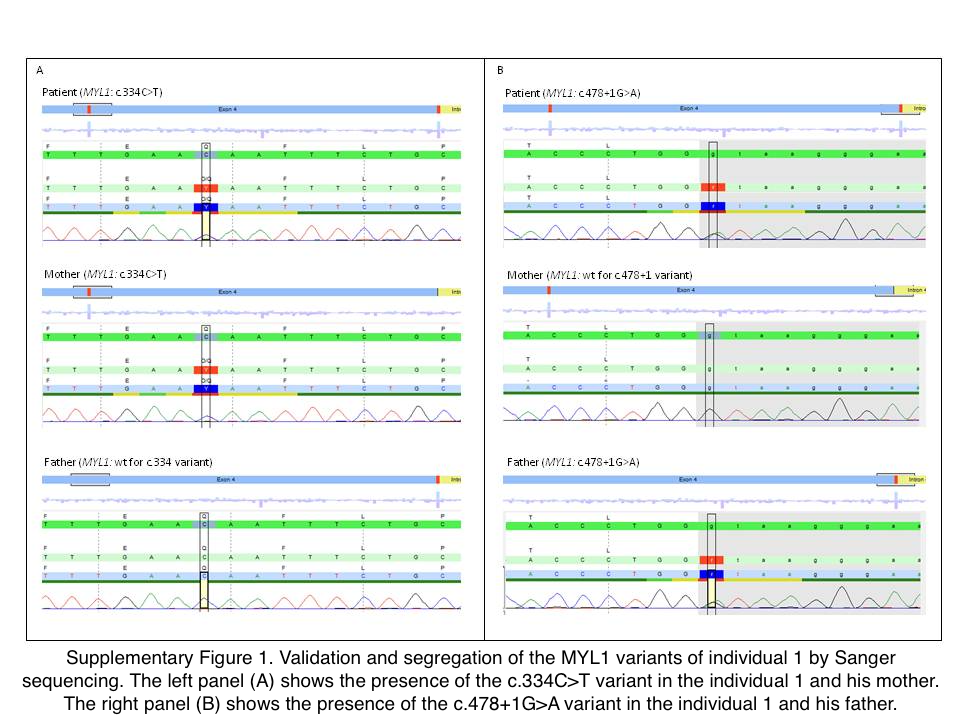

Supplement: Supplementary file 1 — Figure S1 Supporting information. [file NAN-51-e70025-s003.tiff]

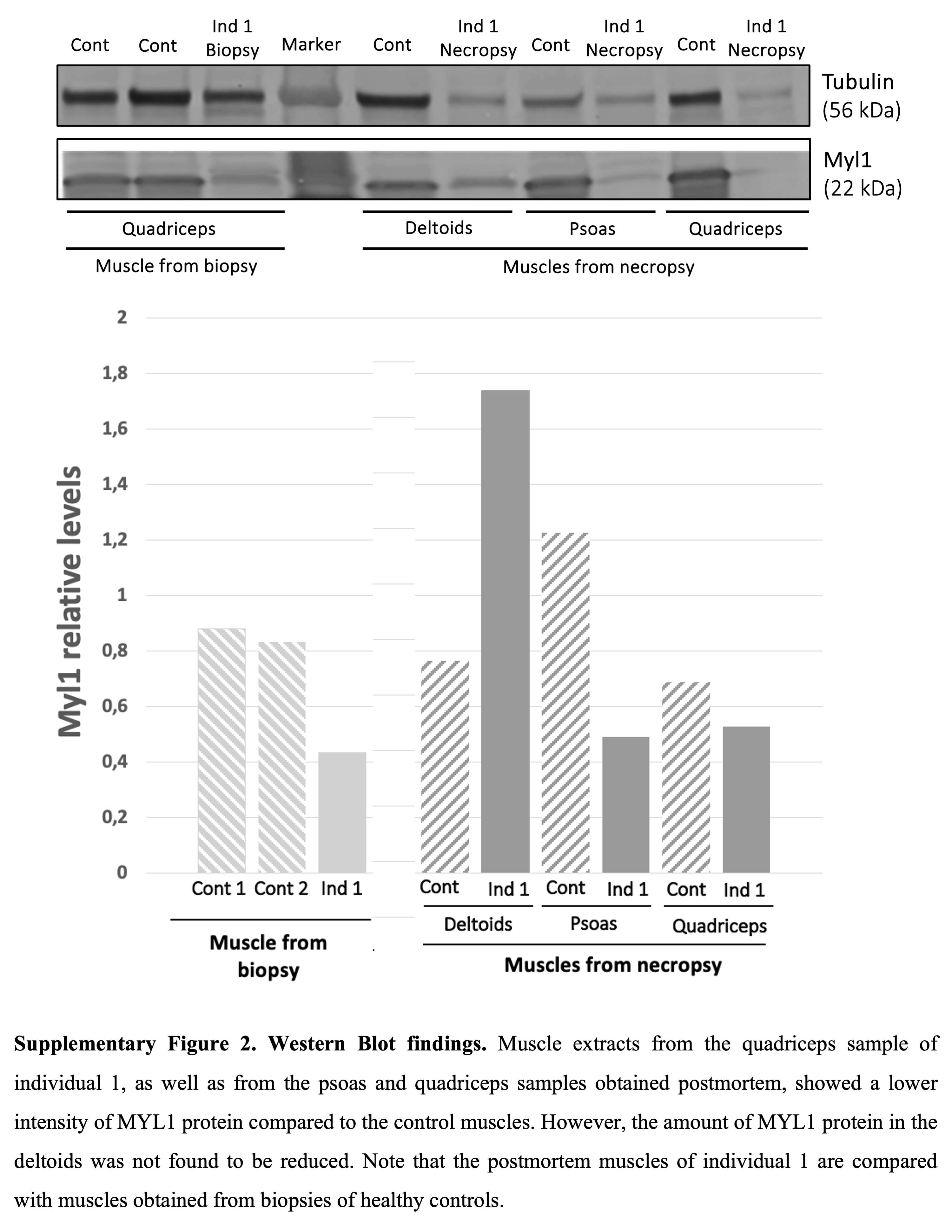

Supplement: Supplementary file 2 — Figure S2 Supporting information. [file NAN-51-e70025-s001.png]
