# Supplementary material for: MYL1‐Related Congenital Myopathy: Clinical, Genetic and Pathological Insights
Source: Neuropathol Appl Neurobiol. 2025 Jun 9;51(3):e70025. doi: 10.1111/nan.70025 (PMC12147433; doi:10.1111/nan.70025)
Supplement: Supplementary file 3 — Table S1 Pathogenic effect of MYL1 variants found in Individuals 1 and 2. [file NAN-51-e70025-s002.docx]

|  | **Gene (Ref. sequence)** | **Nucleotide (amino acid) change** | **gnomAD frequency** | **Segregation** | **Variant classification (ACMG)**  (Last accessed June 2024) | ***In-silico* pathogenicity predictors** | | | | |
| --- | --- | --- | --- | --- | --- | --- | --- | --- | --- | --- |
|  |  |  |  |  |  | **CADD** | **Mutation taster** | **FATHMM-MKL** | **Splice AI** | **NNSplice** |
| **Patient A** | *MYL1* (NM_079420.3) | c.334C>T (p.Gln112Ter) | NR | Compound heterozygosis | LP | 38.0 (Damaging) | 0.81 (Disease causing) | 0.52 (Damaging) | - | - |
|  |  | c.478+1G>A | 0.000003 |  | LP | 34.0 (Damaging) | 0.81  (Disease causing) | 0.91 (Damaging) | 1.0 - Donor loss (Splice altering) | Donor loss (Splice altering) |
| **Patient B** | *MYL1* (NM_079420.3) | c.543del (p.Cys181Ter) | NR | Homozygosis | LP | 33.0 (Damaging) | 0.99 (Disease causing) | - | - | - |

**Supplementary Table 1. Pathogenic effect of *MYL1* variants found in individuals 1 and 2**

Allele frequency in total population (gnomAD v4.1.0, Genome Aggregation Database). Genetic variant classification based on the American College of Medical Genetics and Genomics (ACMG) guidelines, using Franklin (*last accessed June 17, 2024*). *In-silico* pathogenicity predictors used were CADD (Combined Annotation Dependent Depletion; with scores ≥ 20 indicating that the variant is predicted to be among the 1% of the most deleterious substitutions in the human genome), Mutation taster and FATHMM-MKL (with scores ranging from 0 to 1, being 1 the most damaging). Specifically, for the splicing variant: Splice AI (with scores from 0 to 1, predicting the probability that the variant affects splicing at any position within a window of +/- 50bp and with a high precision cutoff of 0.8) and NNSplice (Splice Site Prediction by Neural Network, that compares scores from 0 to 1 for both wild-type and mutated sequences). Abbreviations: NR, not reported; LP, likely pathogenic.
